# Fibroblast growth factor 21 in metabolic syndrome

**DOI:** 10.3389/fendo.2023.1220426

**Published:** 2023-07-27

**Authors:** Ming Yang, Chongbin Liu, Na Jiang, Yan Liu, Shilu Luo, Chenrui Li, Hao Zhao, Yachun Han, Wei Chen, Li Li, Li Xiao, Lin Sun

**Affiliations:** ^1^ Department of Nephrology, The Second Xiangya Hospital of Central South University, Changsha, China; ^2^ Hunan Key Laboratory of Kidney Disease and Blood Purification, Changsha, Hunan, China

**Keywords:** FGF21, metabolic syndrome, obesity, diabetes, hyperlipemia, hypertension

## Abstract

Metabolic syndrome is a complex metabolic disorder that often clinically manifests as obesity, insulin resistance/diabetes, hyperlipidemia, and hypertension. With the development of social and economic systems, the incidence of metabolic syndrome is increasing, bringing a heavy medical burden. However, there is still a lack of effective prevention and treatment strategies. Fibroblast growth factor 21 (FGF21) is a member of the human FGF superfamily and is a key protein involved in the maintenance of metabolic homeostasis, including reducing fat mass and lowering hyperglycemia, insulin resistance and dyslipidemia. Here, we review the current regulatory mechanisms of FGF21, summarize its role in obesity, diabetes, hyperlipidemia, and hypertension, and discuss the possibility of FGF21 as a potential target for the treatment of metabolic syndrome.

## 1 A brief introduction of FGF21

Fibroblast growth factor 21 (FGF21) is a member of the human FGF superfamily, which consists of 22 related proteins from FGF1 to FGF23 (among which FGF15 and FGF19 are homologous proteins in mice and humans, respectively) (1). FGFs consist of a structure-related peptide superfamily of 150-300 amino acids, with a conserved core of approximately 120 amino acids (2). FGFs are a class of polypeptide growth factors that are widely expressed in various organs and tissues and are involved in several processes of cell activity, including cell differentiation, cell proliferation, and embryonic development (3). FGFs play a biological role by binding to four fibroblast growth factor receptors (FGFRs) on the cell membrane. According to their sequences and functional properties, FGFs are classified into seven distinct subfamilies: FGF19, FGF21 and FGF23 belong to the hormone FGF subfamily (4, 5). Members of the hormone FGF subfamily all contain heparin-binding domains that allow them to bind to heparin sulfate proteoglycan, which in turn can initiate the interactions between FGFR and ligand and then activate downstream signaling cascades. However, hormone-type FGFs have a low affinity for FGFR binding, and therefore require the participation of the coreceptor α-Klotho/β-Klotho for effective binding (6, 7). The tissue specificity of α-Klotho/β-Klotho expression restricts the site where hormone-type FGFs play a physiological role (8).

FGF21 was cloned in 2000 by Nobuyuki Itoh’s team (9). Subsequently, in 2005, Kharitonenkov et al. found that FGF21 intervention could effectively reduce plasma glucose and triglyceride levels in ob/ob and db/db mice, and these metabolic regulatory effects could be maintained until at least 24 hours after FGF21 intervention (10). The *FGF21* gene is located on human chromosome 19 and has three exons encoding 209 amino acid residues. The mouse *Fgf21* gene is located on chromosome 7 and encodes 210 amino acid residues (11). Both the human and mouse preprotein of FGF21 contain a 30-amino acid hydrophobic domain that acts as a signal for FGF21 and allows it to be secreted. Mature human FGF21 contains 179 amino acid residues, while in mice, it contains 180 amino acid residues (9, 12). The FGF21 protein is highly conserved. In fact, 75% of its amino acid sequence is shared between mice and humans, and 89% of its amino acid sequence is shared between mice and rats (13, 14). Under physiological conditions, serum FGF21 is mainly secreted by the liver, but other tissues, including adipose tissue, heart, skeletal muscle and the kidney, can also synthesize and secrete FGF21 under certain circumstances (8). After the liver secretes FGF21 into circulation, FGF21 binds to FGFR on the target organ; FGFR, which belongs to the receptor tyrosine kinase (RTK) family (15, 16). Seven major FGFR isoforms have been identified in mammals, namely, 1b, 1c, 2b, 2c, 3b, 3c, and 4 (17–19). Currently, FGFR1c/β-klothos and FGFR3c/β-klothos activation are the major signaling pathways mediating the physiological effects of FGF21 based on cellular receptor activation assays and *in vivo* genetic models (20, 21). When FGF21 binds to receptors on target organs (adipose tissue, liver and muscle), it plays a role in metabolic regulation, including the upregulation of fatty acid β oxidation, ketogenesis and gluconeogenesis (22–24). In addition, adiponectin is a key regulator of metabolic homeostasis (25), and FGF21 has also been shown to strongly induce adiponectin transcription and secretion (26). With the in-depth study of FGF21, its role and molecular mechanism in regulating metabolism have been gradually revealed. Here, we summarize the current research on the role of FGF21 in metabolic syndrome to explore the potential of FGF21 as a potential therapeutic target for metabolic syndrome.

## 2 Transcriptional regulation of FGF21

### 2.1 Peroxisome proliferator-activated receptor α/γ

PPARα is a transcription factor closely involved in metabolic regulation, and PPARα is usually activated during energy deprivation (27). In addition, a variety of drugs, such as fenofibrate, can also induce PPARα activation (28). Fasting has been shown to affect circulating FGF21 levels in the body. While fasting for 2 days did not affect FGF21 levels, the circulating FGF21 levels were 74% higher after 7 days in participants than in control individuals (29). In addition, FGF21 levels were two times higher in non-diabetic patients with hypertriglyceridemia than in the patients in the control group and were 28% higher during fenofibrate treatment (29). Further studies have shown that fasting regulates the FGF21 concentration through the activation of PPARα (29). A similar result was also observed in that participants treated with PPARα agonists showed higher circulating FGF21 levels (30). In addition, the FGF21 levels were very low in PPARα knockout mice in both feeding and fasting states (31). Furthermore, Lundåsen et al. demonstrated that there were PPARα response elements (PPREs) in the promoter region of the mouse and human FGF21 genes, and Inagaki et al. showed that PPARα can directly bind to FGF21 promoters to promote the transcription of FGF21 (32, 33). In addition to PPARα, PPARγ was also identified as a transcription factor of FGF21. Zhou et al. showed that ampelopsin could upregulate insulin sensitivity by activating PPARγ, thereby promoting the expression of FGF21 (34).

### 2.2 Activating transcription factor 4

ATF4, a member of the leucine zipper superfamily, is a multifunctional transcription regulatory protein (35). ATF4 is expressed in most mammalian cell types, and it can be involved in various cellular responses to specific environmental stresses, intracellular disturbances, or growth factors (36). Several studies have also found that ATF4 is involved in the regulation of FGF21 expression under stress. Kim et al. found that mitochondrial dysfunction induced by autophagy defects can promote FGF21 expression by inducing an increase in ATF4 (37). In addition, treatment with a mitochondrial respiratory chain inhibitor also induced FGF21 expression in an ATF4-dependent manner (37). In addition, wogonin, a Scutellaria baicalensis root extract and one of its components, could promote the expression of FGF21, thereby improving metabolic diseases. When the expression of ATF4 was inhibited by ATF4 siRNA, the effect of wogonin on promoting FGF21 and improving metabolism was destroyed (38). Moreover, the TAZ activator TM-25659 increases FGF21 mRNA and protein levels and FGF21 secretion in C2 myotubes by activating the GCN2-phosphoeIF2α-ATF4 signaling pathway, thus reducing fasting blood sugar levels and inflammation (39). These results suggest that ATF4 can mediate changes in the expression of FGF21 and thus regulate metabolic homeostasis in the body under stress. Further studies revealed the molecular mechanism by which ATF4 regulates FGF21 expression. Wan et al. showed that the changes in the expression of FGF21 under endoplasmic reticulum (ER) stress are caused by the binding of ATF4 to the FGF21 promoter, thus promoting the transcription of FGF21 (40). Similar results were also observed in ChIP and luciferase reporter assays, which confirmed that AFT4 can bind to the promoter of FGF21 to promote the expression of FGF21 (41). Moreover, TRIB3 (Tribbles homolog 3), another cellular stress-inducible gene, could inhibit FGF21 expression by binding to ATF4 at the promoter of FGF21 (42). Maruyama et al. showed that there were three response elements for ATF4 in the promoter region of the FGF21 gene: AARE1, AARE2 and AARE3 (43). Under stress conditions, ATF4 regulates the expression of FGF21 by combining with these three response elements.

### 2.3 NFE2-related factor 2

Nrf2 is a major regulator of cell redox status and detoxification response (44). Dozens of protective genes have been identified that are induced in an Nrf2-dependent manner in response to changes in cell redox status (45). In addition to regulating cellular oxidative stress, Nrf2 is also involved in the regulation of cellular metabolism. Genetic or pharmacological activation of Nrf2 can lead to decreased liver lipid levels (46). Moreover, Chartoumpekis et al. demonstrated that Nrf2 knockout mice showed higher plasma levels of FGF21 than the mice in the control group after long-term high-fat feeding (47). Similarly, the levels of mRNA and protein of FGF21 were increased in the livers of Nrf2 knockout mice (48, 49). Furthermore, the luciferase reporter plasmid showed that overexpression of Nrf2 could significantly inhibit FGF21 promoter activity (47). However, different results have been observed regarding the relationship between Nrf2 and FGF21. Intervention with an Nrf2 inducer in db/db mice can effectively upregulate plasma FGF21 levels and hepatic FGF21 expression (50). It seems that Nrf2 positively regulates the expression of FGF21. These different results on the relationship between Nrf2 and FGF21 may be due to different experimental animal models and intervention methods and the role of Nrf2 in regulating FGF21 still needs to be further studied in the future.

### 2.4 Others

**Figure 1:**
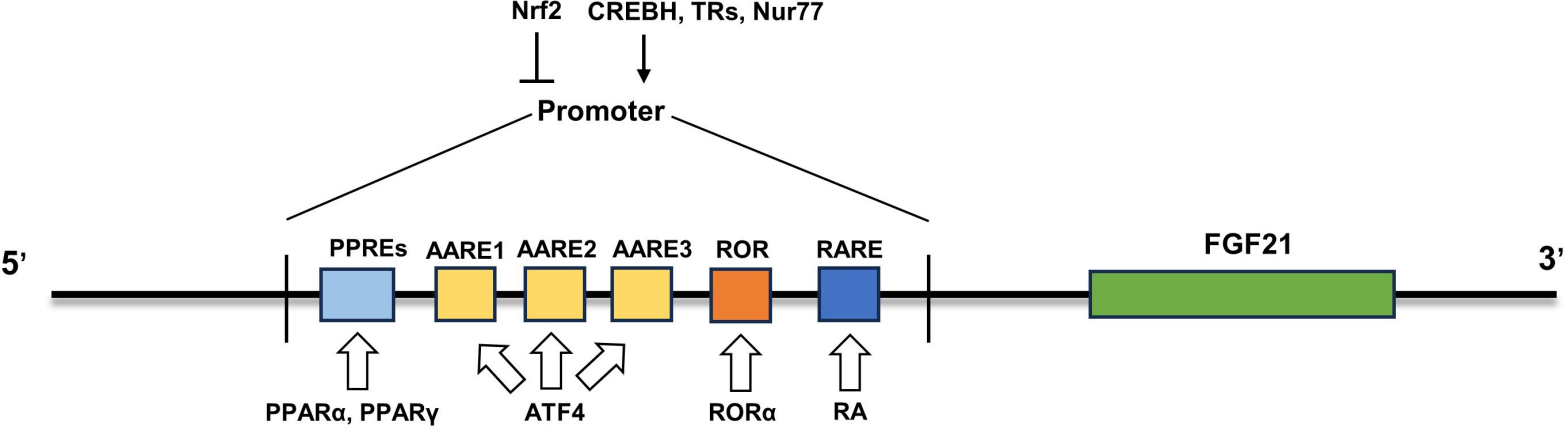
Regulatory mechanism of FGF21 expression. The mRNA expression of FGF21 was upregulated by some transcription factors (PPARα, PPARγ, ATF4, RORα, RA, CREBH, TRs and Nur77) and inhibited by Nrf2. PPARα, peroxisome proliferator-activated receptor α; ATF4, activating transcription factor 4; RORα, retinoic acid receptor-associated receptor α; CREBH, cyclic adenosine monophosphate-responsive element-binding protein H; Nrf2, Nuclear factor erythroid 2-related factor 2; PPREs, PPARα response elements.

In addition to the abovementioned transcription factors, other transcription factors are also involved in the regulation of FGF21 expression in different states. All-trans retinoic acid (RA), the main active metabolite of vitamin A, exerts its regulatory role mainly by binding to the three retinoic acid receptors (RARs) of the nuclear receptor superfamily (51, 52). ob/ob mice treated with RAR agonists showed an anti-obesity phenotype similar to that of mice expressing FGF21 in the liver (53–55). Further studies showed that retinoic acid intervention could promote FGF21 expression by promoting RARβ-FGF21 promoter binding, and this result was also confirmed by adenovirus-mediated RARβ overexpression in the liver, which continuously stimulated liver FGF21 production and secretion (55). Another nuclear receptor, retinoic acid receptor-associated receptor α (RORα), has also been identified to directly regulate the transcription of FGF21. Overexpression of RORα promoted the expression and secretion of FGF21, while inhibition of RORα downregulated it (56). Mechanistically, there is a typical ROR response element in the proximal promoter of the FGF21 gene, to which RORα can bind to promote the transcription of FGF21 (56). Nur77 is a member of the orphan nuclear hormone receptor 4A subgroup, also known as nuclear receptor subfamily 4 group A member 1 (NR4A1) (57). Overexpression of Nur77 increased FGF21 expression *in vivo* and *in vitro*, while inhibition of Nur77 downregulated FGF21 expression (58). Further studies revealed that Nur77 regulates FGF21 expression by binding to the FGF21 promoter (58). In addition, the Src homology 3 domain binding kinase 1 (SBK1)-mediated phosphorylation of Nur77 at serine 344 may promote the translocation of Nur77 to the nucleus for binding to the FGF21 promoter (59). Moreover, the transcription factors cyclic adenosine monophosphate-responsive element-binding protein H (CREBH) (60) and thyroid hormone receptor β (61) can also bind to FGF21 promoters to promote FGF21 expression. Taken together, multiple transcription factors have been identified to modulate the expression of FGF21 under different pathophysiological conditions (**Figure 1**), which sheds light on the strategies to boost FGF21 content and its functions.

## 3 FGF21 and metabolic syndrome

### 3.1 Obesity

Obesity is an important clinical manifestation of metabolic syndrome. Obesity not only causes a variety of metabolic diseases, but also aggravates the progression of metabolic diseases. Therefore, effective weight control is the basis of preventing metabolic diseases. Currently, several studies have shown that FGF21 may be a potential target for obesity treatment. The injection of recombinant human FGF21 into the lateral ventricle of obese mice can effectively increase insulin sensitivity and energy consumption (62). Moreover, FGF21 intervention in diet-induced obesity and ob/ob mice resulted in energy expenditure changes, enhanced fat oxidation, and the inhibition of liver new fat production, thus improving the obesity phenotype (63). Exercise improves the obesity phenotype in part through the effect of FGF21. Geng et al. showed that the expression levels of FGFR1 and β-Klotho were significantly reduced in adipose tissue of mice with high-fat induced obesity. Their expressions levels were effectively restored in adipose tissue by exercise, while these exercise-induced protective effects were blocked by β-Klotho knockout (64). Similarly, overweight and obese men showed significantly higher serum FGF21 levels and significant weight loss after three weeks of high-intensity interval training and high-intensity resistance training compared to individuals in the control group (65). These results suggest that exercise may contribute to weight loss in part by increasing FGF21 secretion and improving FGF21 resistance in adipose tissue.

Natural FGF21 proteins have poor pharmacokinetic properties, and due to their small size, most of them are rapidly eliminated by glomerular filtration, resulting in a short half-life (66, 67). In view of the important role of FGF21 in obesity, the pharmaceutical industry has developed FGF21 analogs or FGF21 receptor agonists to overcome the shortcomings of the natural FGF21 protein, and these treatments have entered the clinical stage. Foltz et al. developed a monoclonal antibody, mimAb1, that specifically activates the β-Klotho/FGFR1c signaling pathway in tissues and obese cynomolgus monkeys showed significant weight loss after mimAb1 intervention (68). Another FGF21 mimetic, LY2405319, also showed significant weight loss in rhesus monkeys (69). FGF21 may reduce body weight through multiple pathways. FGF21 can act on the central nervous system, thereby inducing sympathetic activity and energy expenditure (70). In addition, the increased expression of thermogenic genes, increased total and uncoupled respiration, and enhanced glucose oxidation was also observed in FGF21-treated brown adipocytes (71). Meanwhile, FGF21 also promotes islet beta cell survival and increases insulin sensitivity in peripheral tissues to maintain glucose and lipid homeostasis (72, 73). Therefore, the metabolic regulation mediated by FGF21 analogs may be an effective regimen for weight loss in future studies.

### 3.2 Diabetes mellitus

Diabetes is also an important clinical manifestation of metabolic syndrome. The liver is a key organ of glucose regulation, and FGF21 secreted by the liver has been confirmed to be closely related to the occurrence and development of diabetes. At present, several studies have reported that FGF21 may be a biomarker for the occurrence of diabetes. There were elevated serum levels of FGF21 in patients with diabetes compared with control individuals (74). Moreover, serum FGF21 levels increase during fasting in obese individuals and are associated with insulin resistance (75). Similarly, the plasma FGF21 concentration was positively associated with homeostatic model assessment of insulin resistance (HOMA-IR) in patients receiving lifestyle hypoglycemic therapy only, and patients with higher baseline plasma FGF21 concentrations also had a relatively high risk of glucose progression over a 5-year period (76). FGF21 levels can also be used to assess the effectiveness of diabetes treatment. Liver fat content was increased in newly diagnosed overweight patients with type 2 diabetes combined with nonalcoholic fatty liver disease (NAFLD) compared to individuals in the control group, and was associated with high levels of FGF21. However, hepatic fat content was positively correlated with the relative change in serum FGF21 after 12 weeks of liraglutide treatment (77). The increase in FGF21 levels in diabetes may be due to metabolic disorders and decreased sensitivity to FGF21, so the compensatory synthesis and secretion of more FGF21 by the liver is needed to maintain the metabolic homeostasis of the body. These evidence to show FGF21 may be used as a biomarker in the diagnosis of diabetes.

Moreover, exogenous FGF21 intervention or overexpression of FGF21 can significantly slow the onset of diabetes. Jimenez et al. showed that when treated with FGF21, high-fat diet-fed or db/db mice showed significant improvement in insulin resistance, and inflammation and fibrosis in liver was alleviated (78). A similar result was also observed in that intervention with recombinant human FGF2 alleviated cognitive impairment in obese mice induced by high-fat diet by a regulating impaired glucose tolerance and improving insulin resistance (79).

FGF21 may improve insulin resistance through different pathways. Pan et al. showed that overexpression of FGF21 in the pancreas of db/db mice can effectively improve pancreatic morphology, inhibit β cell apoptosis, and increase glucose stimulation of insulin secretion (80). Mechanistically, FGF21 increases the expression of insulin gene transcription factor and soluble N-ethylmaleimide-sensitive factor attachment protein receptor (SNARE) proteins and activates the phosphatidylinositol 3-kinase (PI3K)/Akt signaling pathway to promote insulin secretion (80). In addition, β-cell-specific knockout of β-klotho (coreceptor of FGF21) led to impaired glucose-stimulated insulin secretion (GSIS) and glucose intolerance in mice, while adenovirus-mediated β-klotho overexpression alleviated the defect of islet GSIS in type 2 diabetic mice (81). Moreover, the insulin-sensitizing protein adiponectin has also been shown to be a downstream effector of FGF21. FGF21 intervention enhanced adiponectin expression and secretion in adipocytes, thereby upregulating circulating adiponectin levels in mice, while the effects of FGF21 on lowering blood glucose and regulating insulin resistance were partially inhibited when adiponectin was knocked out (82). Similarly, FGF21 could effectively lower blood glucose levels and enhance insulin sensitivity in ob/ob mice and diet-related obese mice only when adiponectin function was present (83). In addition, the overexpression of FGF21 in the liver can upregulate the expression of genes involved in fatty acid oxidation, thus accelerating energy expenditure and reducing steatosis (84). This could also benefit the treatment of diabetes.

In addition to the manifestations of hyperglycemia and insulin resistance, a variety of microangiopathies can also be caused by diabetes. These microangiopathies result in damage to target organs, such as diabetic cardiomyopathy (85), diabetic nephropathy (86) and diabetic retinopathy (87). FGF21 has also been reported to have a protective effect against diabetes complications. FGF21 knockout diabetic mice showed earlier and more severe cardiac dysfunction, remodeling, and oxidative stress (88) and the effective delivery of FGF21 to myocardial tissue through the new drug delivery system can reduce myocardial hypertrophy, cell apoptosis and interstitial fibrosis in diabetic mice (89). Moreover, Jin et al. showed that treadmill exercise alleviated diabetes-induced cardiac dysfunction in mice by upregulating FGF21 sensitivity. Mechanistically, FGF21 activates the AMPK/FOXO3/SIRT3 signaling pathway, thereby enhancing mitochondrial function and improving diabetic myocardial injury (90). In addition, FGF21 ameliorates myocardial damage in diabetes by activating AMPK-AKT2-Nrf2-mediated antioxidant pathways and AMPK-ACC-CPT1-mediated lipid-lowering effects (91). FGF21 is also essential in slowing the progression of diabetic nephropathy. Higher serum FGF21 levels were inversely associated with the glomerular filtration rate in patients with diabetes (92). However, the increased level of FGF21 in diabetic nephropathy patients may be due to the increased compensatory secretion of FGF21 in the body due to the presence of FGF21 resistance. However, because FGF21 is mainly excreted by the kidney, FGF21 excretion is reduced and therefore retained in the body when renal function is damaged. Cheng et al. demonstrated that fenofibrate could reduce renal oxidative stress and inflammation by upregulating the expression of FGF21 and activating the Nrf2 signaling pathway in the diabetic state, and these protective effects were eliminated in FGF21-deficient mice (93). Similarly, recombinant human FGF21 intervention significantly reduced the urinary albumin/creatinine ratio (ACR) and inhibited renal mesangial dilation, thereby alleviating diabetic kidney injury in db/db mice (94).

Although FGF21 has shown a protective effect in the treatment of diabetes, the effect of FGF21 and its analogs on lowering blood glucose levels is still not obvious in current clinical trials. This may be related to the resistance of FGF21 in the long-term course of diabetes. In the future, treatment with FGF21 in combination with drugs or exercise to improve FGF21 resistance may be a potential approach for the treatment of diabetes and its complications. In addition, FGF21 may also be used as a biomarker in the diagnosis of diabetes in the future.

### 3.3 Hyperlipemia

Hyperlipidemia is also a common clinical manifestation of metabolic syndrome. After strictly matching the BMI of subjects, serum FGF21 levels were positively correlated with serum total cholesterol, triglyceride and LDL cholesterol levels. The most significant correlation was between FGF21 and triglycerides, and FGF21 levels were independently associated with pericardial fat volume (95). In addition, Liu et al. showed that recombinant FGF21 intervention reduced cholesterol levels by promoting brown adipose tissue (BAT) activation and white adipose tissue (WAT) browning, thereby enhancing fatty acid uptake into BAT and brown WAT for metabolism (96). Moreover, dietary protein dilution upregulated the expression of FGF21 and accelerated the oxidative utilization of fatty acids in tissues, thus effectively alleviating hypertriglyceridemia and fatty liver (97). In addition to accelerating the oxidation of fatty acids, FGF21 can also reduce cholesterol synthesis and reduce hypercholesterolemia by inducing adiponectin production in adipose tissue, which inhibits the transcription factor cholesterol regulatory element binding protein-2 (98). Similarly, restoring the function of FGF21 can inhibit the expression of SREBP-1c and thus inhibit lipid synthesis in the liver and upregulate the expression of adipose triglyceride lipase (ATGL) and hormone-sensitive lipase (HSL) in WAT to promote lipolysis (99). Moreover, FGF21 treatment significantly reduced plasma levels of nonesterified fatty acids (NEFAs) and hepatic triglyceride (TG). Furthermore, FGF21 also promoted the catabolism of TG-rich lipoproteins in white adipose tissue and brown adipose tissue (100). Nowadays, there are some clinical trials to reveal the role of FGF21 in reducing lipid levels in patients. Treatment of overweight/obese type 2 diabetic patients with the long-acting FGF21 analogue (PF-05231023) significantly reduced circulating triglyceride and low-density sterol levels and increased high-density lipoprotein and adiponectin levels (101). Similarly, several other FGF21 analogues are also effective in lowering lipid levels in patients (102–104). These findings strongly support the lipid-lowering effects of FGF21. However, the molecular mechanisms and targets of FGF21 should be further elucidated to better avoid its potential side effects.

### 3.4 Hypertension and atherosclerosis

**Figure 2:**
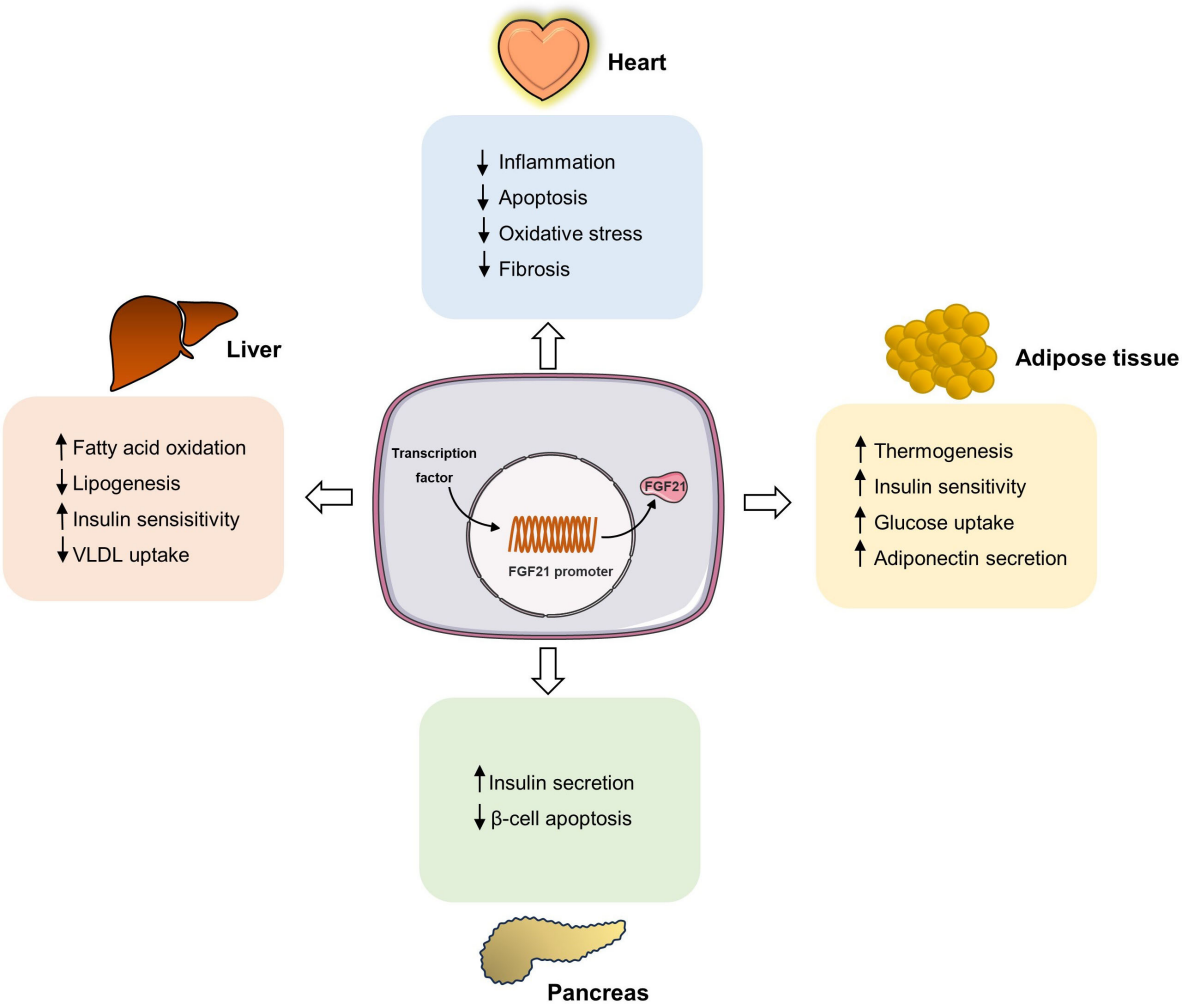
FGF21 regulates intracellular metabolic homeostasis in different tissues. Multiple transcription factors can stimulate the transcriptional expression of FGF21, and FGF21 circulates to the target organ to bind to the receptors. In adipose tissue, FGF21 increases thermogenesis, upregulates insulin sensitivity, glucose uptake ability, and increases adiponectin secretion. In heart, FGF21 inhibits inflammation, apoptosis, and fibrosis. In liver, FGF21 enhances fatty acid oxidation and insulin sensitivity, while inhibits VLDL uptake and lipogenesis. Moreover, FGF21 can increase insulin secretion and inhibit islet β cell apoptosis.

*FGF21* mRNA levels were higher in hypertensive patients than in healthy control individuals (105). Similarly, there were increased serum levels of FGF21 in elderly patients with hypertension and carotid atherosclerosis, and FGF21 levels can be used to diagnose carotid atherosclerosis and predict prognosis (106). The association between FGF21 and hypertension has also been observed in animal models. FGF21 levels were significantly increased in the liver, heart, and serum in a mouse model of angiotensin II-induced hypertension compared to the control group (107). Additionally, FGF21 knockout mice developed more severe hypertensive heart disease, characterized by increased cardiac dysfunction and fibrosis (107). FGF21 ameliorates hypertension and target organ damage through different signaling pathways. Pan et al. also showed that a lack of FGF21 exacerbates angiotensin II-induced hypertension and vascular dysfunction, and this adverse effect can be reversed by FGF21 supplementation (108). Mechanistically, FGF21 acts on kidney and adipose tissue angiotensin-converting enzyme 2 (ACE2) by converting angiotensin II to angiotensin-(1-7), thereby inhibiting hypertension and alleviating vascular damage (108). The protective effects of FGF2 were partially blocked by ACE2 deficiency (108). Moreover, the protective effects of FGF21 on cardiac hypertrophy, fibrosis, and apoptosis were suppressed by SIRT1 elimination in Ang II-induced hypertensive mice (109). Further studies showed that FGF21 could significantly upregulate the activity of SIRT1 deacetylase, further activate the AMPK signaling pathway, change the transcriptional activities of FoxO1 on its downstream target genes catalase (Cat), MnSOD (Sod2) and Bim, and finally, inhibit the accumulation of reactive oxygen species to reduce heart injury (109). In addition, Refined-JinQi-JiangTang tablets reduce hypertension by activating the FGF21/FGFR1 signaling pathway (110). These results suggest that FGF21 may also be a potential target for hypertension treatment (**Figure 2**). Atherosclerosis is also an important manifestation of a metabolic disorder. In a clinical study involving 253 patients, elevated serum FGF21 levels were reported as an independent risk factor for coronary artery disease (111). Another study involving 670 patients also demonstrated a positive association between serum FGF21 levels and carotid atherosclerosis, independent of lipid levels (112). This implies that FGF21 can be used as a biomarker of atherosclerosis. Moreover, studies have also shown that increasing the expression of FGF21 can ameliorate atherosclerosis. Sappanwood extract could regulate the FGF21/SREBP-2 signaling pathway to alleviate lipid metabolism disorders and atherosclerosis in rats (113). Moreover, Li et al. found that aerobic exercise may increase the sensitivity of FGF21 to inhibit the development of atherosclerosis (114). A similar result was also observed in that treatment with exogenous FGF21 notably reduced the aortic sinus plaque area of ApoE^-/-^ mice (115). In addition, FGF21 can also reduce atherosclerotic lesion severity by accelerating the turnover of triglyceride-rich lipoproteins by activating the brown adipose tissue and browning of white adipose tissue (96).

### 3.5 NAFLD

NAFLD is also a common metabolic disorder, and it is estimated that more than 1 billion people have NAFLD worldwide (116). Several studies have shown that FGF21 levels were significantly lower in patients with NAFLD than in control individuals (117–119). Liver-specific overexpression of FGF21 attenuated HFD-induced lipotoxicity in mice. Furthermore, hepatic FGF21 overexpression ameliorated hyperglycemia and hypertriglyceridemia by activating thermogenic tissues and reducing adipose tissue inflammation (75). Moreover, the inhibition of FGF21 expression also promoted the transformation of nonalcoholic steatohepatitis to hepatitis (120). In addition, a clinical trial evaluating the safety and efficacy of efruxifermin, a long-acting Fc-FGF21 fusion protein, in nonalcoholic steatohepatitis showed that efruxifermin intervention significantly reduced the hepatic fat fraction in patients with stage F1-F3 nonalcoholic steatohepatitis with an acceptable safety profile (121). Moreover, a PEGylated human fibroblast growth factor 21 (FGF21) analogue pegbelfermin has also been shown to significantly reduce liver fat fraction in patients with nonalcoholic steatohepatitis (102). Similarly, obese patients with mild hypertriglyceridemia had significant improvements in lipid levels and liver fat mass and biomarkers of liver injury with LLF580 (an FGF21 analog) treatment every 4 weeks (122). These results suggest that targeting FGF21 may be a potential strategy for the treatment of NAFLD.

## 4 Conclusion and future prospective

Here, we summarize recent studies on the relationship between FGF21 and metabolic syndrome (obesity, diabetes, hyperlipidemia, and hypertension), confirming the importance of FGF21 in regulating metabolic syndrome. On the one hand, FGF21 can be used as a biomarker to predict the occurrence and prognosis of metabolic disorders in the early stage. On the other hand, exogenous supplementation with FGF21 has also been proven to be effective in relieving metabolic disorders. Although the results were surprising, the use of FGF21 is as a treatment for metabolic syndrome still requires further investigation. Natural FGF21 has poor pharmacokinetics, so it needs to be modified to better play its role. Currently, the development of FGF21 analogues or mimics through biopharmaceutical engineering approaches also greatly enriches the possibility of FGF21 as a therapeutic target for metabolic diseases, such as the clinical trials of LY2405319 and PF-05231023 in metabolic diseases are under way. In addition, FGF21 can activate a variety of signaling pathways in the body, and its side effects need to be further clarified. Moreover, the current research on FGF21 and metabolic syndrome is mostly in the basic experimental stage, and its clinical research needs to be further strengthened. Although many aspects still need to be addressed, FGF21 is still an ideal target for the treatment of metabolic syndrome.

## Author contributions

MY the first draft of the manuscript. CBL, NJ, YL, SL, HZ, CRL, YH, WC, LL, LX, provided consultations on the preparation of the work. LS contributed to manuscript revision, read, and approved the submitted version.
